# Quantification of bell-shaped size selectivity in shrimp trawl fisheries using square mesh panels and a sorting cone after a Nordmøre grid

**DOI:** 10.1371/journal.pone.0222391

**Published:** 2019-09-12

**Authors:** Manu Sistiaga, Bent Herrmann, Roger Bertrand Larsen, Jesse Brinkhof

**Affiliations:** 1 SINTEF Ocean, Brattørkaia, Trondheim, Norway; 2 Institute of Marine Research, Bergen, Norway; 3 The Arctic University of Norway, UiT, Breivika, Tromsø, Norway; MARE – Marine and Environmental Sciences Centre, PORTUGAL

## Abstract

Shrimp trawlers in the Barents Sea use a Nordmøre sorting grid ahead of a small-mesh codend to avoid bycatch while catching shrimps efficiently. However, small fish can still pass through the grid to enter the codend, which increases their risk of being retained. In this study, we quantified the selectivity of a standard Nordmøre grid used together with one of two different codend designs, namely a diamond mesh codend with square mesh panels and a codend with a square mesh sorting cone section, for deep-water shrimp (*Pandalus borealis*), redfish (*Sebastes* spp.), and American plaice (*Hippoglossoides platessoides*). For the first time, the selective properties of these two alternative designs were estimated and compared to those of a Nordmøre grid used together with a 35-mm diamond mesh codend, which is the compulsory gear used in the fishery today. With this traditional codend, the size selectivity of both bycatch species showed the expected characteristic bell-shaped size selection pattern, with low retention probability of very small fish and bigger fish but with high retention probability of certain sizes of juveniles. Using the square mesh sorting cone significantly reduced the maximum retention risk of redfish. The maximum retention with the diamond mesh codend with square mesh panels was estimated to be 14% lower than that of the traditional codend, but the difference was not statistically significant. The two alternative codend designs did not result in any significant reduction in bycatch of American plaice.

## Introduction

The deep-water shrimp *Pandalus borealis* is a commercially important species that has been widely fished in the Northeast Atlantic for the past four decades [1]. In the Barents Sea, specifically, the catches of this species have oscillated between ~20,000 and ~40,000 tons since 2010 [2]. However, bycatch of juvenile fish of species like redfish (*Sebastes* spp.), cod (*Gadus morhua*), haddock (*Melanogrammus aeglefinus*), American plaice (*Hippoglossoides platessoides*) and Greenland halibut (*Reinhardtius hippoglossoides*) remains a problem in this area [3, 4, 5]. For example, according to estimates presented by the International Council for Exploration of the Sea (ICES), in the last decade up to 110 million redfish have been discarded from the Barents Sea shrimp trawl fishery every year [6]. The bycatch problem is related to the small mesh size used in the shrimp trawl (minimum 35 mm), which can retain large numbers of fish and other bycatch when they are abundant on the fishing grounds. The introduction of the Nordmøre grid in the early 1990s eliminated the bycatch of larger fish, as they would not be able to pass through the grid and into the trawl codend [7]. This grid is used in many fisheries around the world [1, 8, 9], and, in the Barents Sea, its use (with minimum bar spacing of 19 mm) became compulsory in 1993. Although the Nordmøre grid eliminates most of the bycatch entering a shrimp trawl, juvenile fish are still able to pass through the grid and enter the codend together with the targeted shrimp. The species and numbers of juveniles retained can vary greatly, from a few individuals of a single species to hundreds of individuals of multiple species per haul, depending on the area and season.

Today, several decades after the introduction of the Nordmøre grid in the shrimp trawl fishery, concerns remain about the bycatch risk of juveniles of several fish species. In particular, redfish species, which are slow growing and whose stocks in the Northeast Atlantic are at challenging levels, are of special concern [10]. The current regulations in the Northeast Atlantic shrimp fishery allow retention of a few individuals of each of the regulated commercial species per 10 kg of catch. For redfish, for example, the limit is set at three individuals per 10 kg of shrimp [11]. If the catch exceeds this number, the authorities close the area to shrimp trawlers [12]. In addition to the bycatch species, excessive catches of undersized shrimp can lead to area closures. In this respect, the regulation in the Barents Sea states that the shrimp catch cannot contain more than 10% of undersized (i.e. < 15 mm carapace length) individuals. Area closures can have drastic implications for fishermen, as they can last for weeks and restrict access of trawlers to good shrimp-fishing grounds.

Bycatches of juvenile fish other than the regulated species do not lead to problems such as area closures, but they have important implications for the shrimp fishery. Apart from causing additional work and practical problems with sorting the catch onboard, the environmental impact of harvesting fish only to discard them needs to be considered. American plaice is one of the most abundant non-regulated bycatch species captured in the Northeast Atlantic shrimp fishery. Due to its morphology, the size range of individuals that can pass through the grid is large and depends on the orientation of the fish when it meets the grid. If the fish is optimally orientated towards the grid and makes selectivity contact [13, 14] with it, individuals of up to ~35 cm can pass through the grid [4].

The selectivity system used by the shrimp trawlers in the Barents Sea is a dual system. The first selection process takes place at the Nordmøre grid, and the individuals not sorted out by the grid undergo a second selection process in the codend [4]. Numerous attempts have been made to improve selectivity by modifying the grid section, but no major performance breakthroughs with respect to the original grid design have been reported [3, 15]. Therefore, efforts to improve selectivity now are directed to potential changes in the codend. The objective is for the codend to sort out at least part of the bycatch fish species and undersized shrimp that pass through the grid. Today, the fleet is required to use codends with a minimum mesh size of 35 mm, and most vessels in the fishery use diamond mesh codends. This mesh size was established in the late 1960s and has not been changed since then [16]. Larsen et al. [4] concluded that "*fish within a limited size range and undersized shrimps retained in the 35-mm codend will continue to be a problem for the northern shrimp fleet*".

Different codend constructions with square meshes have been shown to have good selective properties for finfish and are applied in many fisheries [17, 18]. These types of construction are not widespread in shrimp fisheries, but several studies have successfully applied square mesh panels/codends in shrimp trawl fisheries [19, 20, 21]. Therefore, testing codend constructions that incorporate square mesh size selectivity and comparing them to the 35-mm diamond mesh codends used by the fleet today is relevant. Due to the morphological characteristics of redfish, we would expect the use of square mesh constructions in the codend would increase the escape of undersized individuals of this species. However, the effect of using square meshes regarding release of flatfish species including American plaice would be more doubtful due to their morphology compared to the shape of square meshes [22, 23, 24].

The main objective of the present study was to determine whether the selectivity of shrimp and redfish could be improved by changing the traditional diamond mesh codend used in the fishery today to a different codend design [25]. Further, we investigated whether these design changes could influence the selectivity of flatfish species such as American plaice. Two new codends were tested: a diamond mesh codend with square mesh side panels and a square mesh sorting cone followed by a blinded codend. Specifically, we aimed to answer the following research questions:

- do grid and codend data collected with codends other than diamond mesh codends exhibit a bell-shaped size selection?
- does a diamond mesh codend with square mesh panels or a square mesh sorting cone followed by a blinded codend improve size selectivity for redfish or undersized deep-water shrimp compared with a diamond mesh codend?
- do these design changes affect the selectivity of American plaice?

## Materials and methods

### Vessel, area, time, and gear set-up

Full-scale tests of the gear were performed on board the R/V "Helmer Hanssen" (63.8 m LOA and 4080 HP) from 16 to 28 February 2016. The fishing grounds were located east of Hopen Island in the northern Barents Sea (76^o^06,6–76^o^04,0N and 35^o^37,5–35^o^07,6E). The trawl gear used was composed of Thyborøn T2 (6.5 m^2^ and 2200 kg each) trawl doors, 40 m double sweeps, 19.2 m long rockhopper gears (built of three sections with 46 cm rubber discs), and two identical Campelen 1800# shrimp trawls (40–80 mm meshes in the wings and belly (2-mm polyethylene twine)) that were towed one at the time. The Campelen trawl has a 19.2 m fishing line and a wingspread and height of 15 m and 6.5 m, respectively, when the door distance is at ca. 50 m. We kept the distance between the doors between 48 and 52 m during the tows using a 20 m long “door distance restrictor rope” that was linked between the warps 80 m in front of the doors (more detailed information regarding the gear can be found in Larsen et al. [4]). In each of the Campelen trawls a 4-panel Nordmøre grid section was installed between the belly section and the codend. The grids in the sections were made of stainless steel and were 1500 mm high and 750 mm wide. They were mounted so that they would maintain an angle of 45° while fishing.

**Fig 1:**
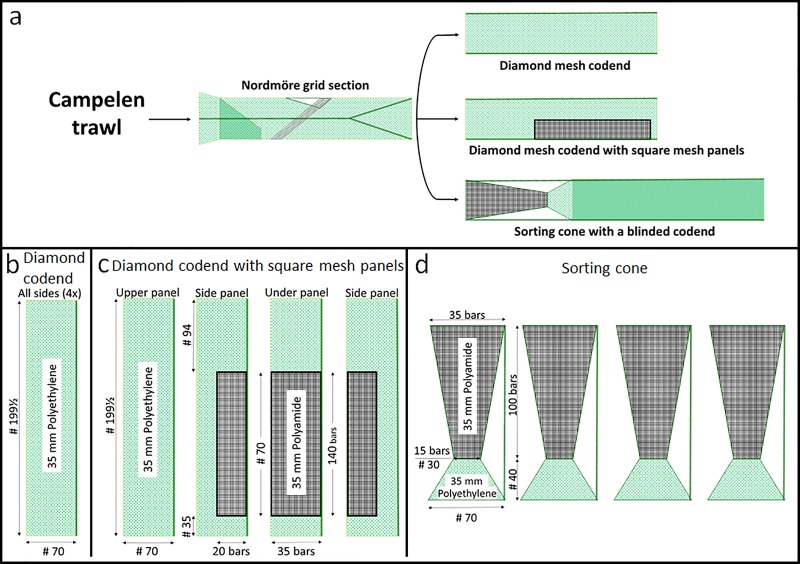
Schematic view of the three gear configurations tested during the experiments. (a): diamond mesh codend (b), diamond mesh codend with square mesh panels (c) and square mesh sorting cone with a blinded codend (d).

In this study, we present data collected with three different gear configurations. The Nordmøre grid section in all three gears was identical, but the codend was different in each configuration: a 35-mm diamond mesh codend (mesh size 33.8 ± 1.0 (mean ± SD)), a 35-mm diamond mesh codend with square mesh panels (mesh size 32.2 ± 0.1 mm, full mesh size = 2 x mesh bar length), and a square mesh sorting cone (mesh size 26.3 ± 0.9, full mesh size = 2 x mesh bar length) followed by a blinded codend (Fig 1). We installed a blinded codend after the sorting cone because we wanted to evaluate the sorting properties of the sorting cone alone.

**Fig 2:**
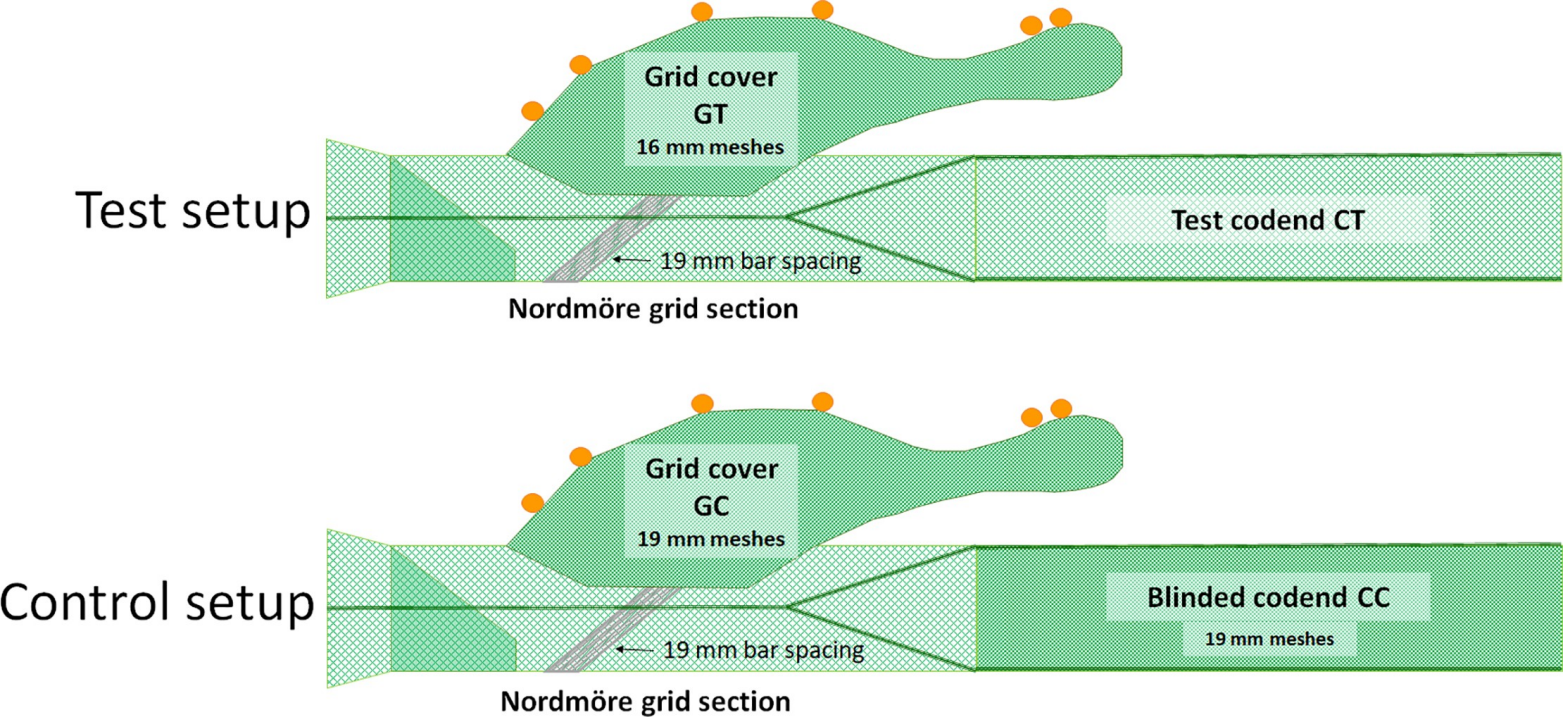
Experimental gear setups used during the trials. Test setup (top) and control setup (bottom).

During the trials we used two experimental setups, a test setup and a control setup (Fig 2). The bar spacing in the grids and the mesh size in the codends used in both setups were measured using a procedure described in Wileman et al. [26]. The grid in the test setup was measured to be 18.8 ± 1.2 mm (mean ± SD), whereas the grid in the control setup was 18.8 ± 0.4 mm. In both setups, the grid was covered with a small mesh size cover to capture the fish and shrimp escaping through the escape outlet of the grid. The covers used were of the type described in Wileman et al. [26] and the same as those used in several other experiments including Larsen et al. [4]. The meshes in the covers of the test and control setup were measured to be 16.4 ± 0.5 mm and 18.9 ± 1.2 mm, respectively. Despite a slight difference in the mesh size, both covers were installed with low hanging ratios (E) (E = 0.1–0.2) and assumed to be equally non-selective for the shrimp and bycatch sizes found in the experiments. In the test setup, we shifted between the three gear configurations tested (Fig 1), whereas in the control setup we used a codend with an inner net (mesh size 18.5 ± 0.9 mm) that blinded the codend during the whole trial period. This inner net was installed with a low hanging ratio to retain all sizes of shrimp and fish juveniles that entered the trawl.

Test and control hauls were all conducted in the same fishing area and during the same cruise. The catch data from these groups of hauls were applied to estimate the size selectivity for shrimp, redfish, and American plaice for each of the three gear configurations tested. For the test hauls, the catch was collected in the test grid cover (GT) and in the test codend (CT), whereas for control hauls, the catch was collected in the control grid cover (GC) and in the control codend (CC) (Fig 2). For each haul, the catch was first sorted by species, and then the lengths of individuals were measured. All fish in the cover and the codend were measured, whereas from the shrimp catch a subsample of ca. 1 kg was taken for measurements. The bycatch fish species were sorted into 1-cm length groups using a measuring board, whereas the shrimp were measured in 1-mm wide length groups using calipers. Thus, the catch data consisted of count numbers (*n*) of individuals per length class of the different species collected in each of the compartments.

The trial period and data used in the present study partly overlapped with the study of the Nordmøre grid and a 35-mm codend carried out by Larsen et al. [4]. However, Larsen et al. [4] only reported results for the baseline codend (35-mm diamond mesh), whereas in the present study we report results for the two alternative codends: a diamond mesh codend with square mesh panels and a codend with a square mesh sorting cone.

### Data analysis and parameter estimation

The procedure for analyzing the selectivity data collected for each of the three trawl configurations followed the method described by Larsen et al. [4]. Thus, the overall (*r*_*combined*_(*l*,***υ***_*grid*_,***υ***_*codend*_)) and individual size selection for the Nordmøre grid (*p*_*grid*_(*l*,***υ***_*grid*_)) and codend (*r*_*codend*_(*l*,***υ***_*codend*_)) were described by the model:

$$\begin{array}{c} r_{combined}(l,\upsilon_{grid},\upsilon_{codend})=p_{grid}(l,\upsilon_{grid})\times r_{codend}(l,\upsilon_{codend})\\ p_{grid}(l,\upsilon_{grid})=C_{grid}\times \left(1.0-logit(l,{L50}_{grid},{SR}_{grid})\right)\\ r_{codend}(l,\upsilon_{codend})=logit(l,{L50}_{codend},{SR}_{codend}) \end{array}$$ (1)

where *l* denotes the length of shrimp, redfish, or American plaice and *p*_*grid*_(*l*,***υ***_*grid*_) is the length-dependent passage probability through the Nordmøre grid. The length-dependent passage probability through the Nordmøre grid considers that some shrimp or fish may not contact the grid at all or do so with such a poor orientation that they will not be subjected to a length-dependent probability of passing though. This is modeled by the length-independent parameter *C*_*grid*_. For a fish or shrimp contacting the grid with sufficiently good orientation to provide a length-dependent chance of passing through it, Eq (1) assumes the traditional *logit* size selection model with the parameters *L*50_*grid*_ and *SR*_*grid*_ (see Wileman et al. [26]). For the codend size selection, Eq (1) assumes that the retention probability can be modeled by a *logit* model with the parameters *L*50_*codend*_ and *SR*_*codend*_.

To estimate the average size selection of the Nordmøre grid and the specific codend in the test trawl, we paired the pooled catch data from the test hauls with the pooled catch data from the control hauls. Based on this approach, the experimental data in the analysis were treated like three compartment data. Shrimp, redfish, and American plaice caught were observed in GT, CT, or (GC + CC). For the estimation based on the size-selection model (1), we needed to express the probabilities *p*_*GT*_, *p*_*CT*_, and *p*_*GC+CC*_ for shrimp or fish of a specific length *l* that would be observed in each of these three compartments, conditioned they were caught (Larsen et al. [4]):

$$\begin{array}{c} p_{GT}(l,\upsilon_{grid},\upsilon_{codend},SP)=\frac{SP\times (1.0-p_{grid}(l,\upsilon_{grid}))}{1.0+SP\times (r_{combined}(l,\upsilon_{grid},\upsilon_{codend})-p_{grid}(l,\upsilon_{grid}))}\\ p_{CT}(l,\upsilon_{grid},\upsilon_{codend},SP)=\frac{{SP\times r}_{combined}(l,\upsilon_{grid},\upsilon_{codend})}{1.0+SP\times (r_{combined}(l,\upsilon_{grid},\upsilon_{codend})-p_{grid}(l,\upsilon_{grid}))}\\ p_{GC+CC}(l,\upsilon_{grid},\upsilon_{codend},SP)=\frac{1.0-SP}{1.0+SP\times (r_{combined}(l,\upsilon_{grid},\upsilon_{codend})-p_{grid}(l,\upsilon_{grid}))} \end{array}$$ (2)

*SP* is the split factor that quantifies the probability that a shrimp, redfish, or American plaice enters the selective section in one of the test hauls with the specific codend, provided it enters into either one of these test hauls or one of the control hauls. *SP* is traditionally accounted for in paired-gear data analysis [26]. By using Eq (2), the values for the parameters in selection model (1) can be estimated from the collected experimental data by minimizing the following function with respect to ***υ***_*grid*_,***υ***_*codend*_, and *SP* [4]:

$$\sum_{i=1}^{a}\sum_{l}\left\{\frac{{nGT}_{li}}{{qGT}_{i}}\times ln(p_{GT}(l,\upsilon_{grid},\upsilon_{codend},SP))+\frac{{nCT}_{li}}{{qCT}_{i}}\times ln(p_{CT}(l,\upsilon_{grid},\upsilon_{codend},SP))\right\}+\sum_{j=1}^{b}\sum_{l}\left\{\left(\frac{{nGC}_{lj}}{{qGC}_{j}}+\frac{{nCC}_{lj}}{{qCC}_{j}})\times ln(p_{GC+CC}(l,\upsilon_{grid},\upsilon_{codend},SP)\right)\right\}$$ (3)

where the inner summations are over length classes *l* in the experimental data and the outer summations are over experimental fishing hauls *i* (from *1* to *a*) and *j* (from *1* to *b*) with, respectively, the specific test codend and control setup. *nGT*_*li*_, *nCT*_*li*_, *nGC*_*lj*_, and *nCC*_*lj*_ are the number of shrimp or fish length measured belonging to length class *l* in haul *i* and *j* in the respective compartment. *qGT*_*i*_, *qCT*_*i*_, *qGC*_*j*_, and *qCC*_*j*_ are subsampling factors that quantify the fraction of the caught individuals being length-measured in the respective compartments in the individual hauls. Minimizing (3) with respect to the parameters in it is the same as maximizing the likelihood for the observed experimental data based on a multinomial model, assuming that model (1) describes the experimental data sufficiently well. The observed experimental length-dependent portioning of the catches between the three compartments GT, CT, and GC + CC, which model (2) is expected to describe, are given by Larsen et al. [4]:

pGTl^=∑i=1a(nGTliqGTi)∑i=1a(nGTliqGTi+nCTliqCTi)+∑j=1b(nGCljqGCj+nCCljqCCj)pCTl^=∑i=1a(nCTliqCTi)∑i=1a(nGTliqGTi+nCTliqCTi)+∑j=1b(nGCljqGCj+nCCljqCCj)pGC+CCl^=∑j=1b(nGCljqGCj+nCCljqCCj)∑i=1a(nGTliqGTi+nCTliqCTi)+∑j=1b(nGCljqGCj+nCCljqCCj) (4)

Due to the experimental procedure followed, there was no obvious way to pair the data from the individual test and control hauls. Hence, to estimate the mean selectivity parameters for the experimental gear, the expected length-dependent total catches for the test hauls were combined and compared with the expected combined total catches for the control hauls as formulated in function (3). The confidence limits for the parameters and curves for the size selection model were estimated using a double bootstrapping method that accounts for the uncertainty resulting from this unpaired nature of the data [4]. We performed 1000 bootstrap repetitions to calculate the 95% percentile confidence limits [27, 28] for the selection parameters and curves.

The model’s ability to describe the experimental data sufficiently well was evaluated based on the p-value, model deviance *versus* degrees of freedom (DOF), and inspection of how the model curve reflects the length-based trend in the data [26]. The p-value expresses the likelihood of obtaining at least as big a discrepancy between the fitted model and the observed experimental data by coincidence. The analysis was carried out using the software SELNET [29, 30, 31], which implements the models and the bootstrap method described above.

### Indicators for bell-shaped retention probability

**Fig 3:**
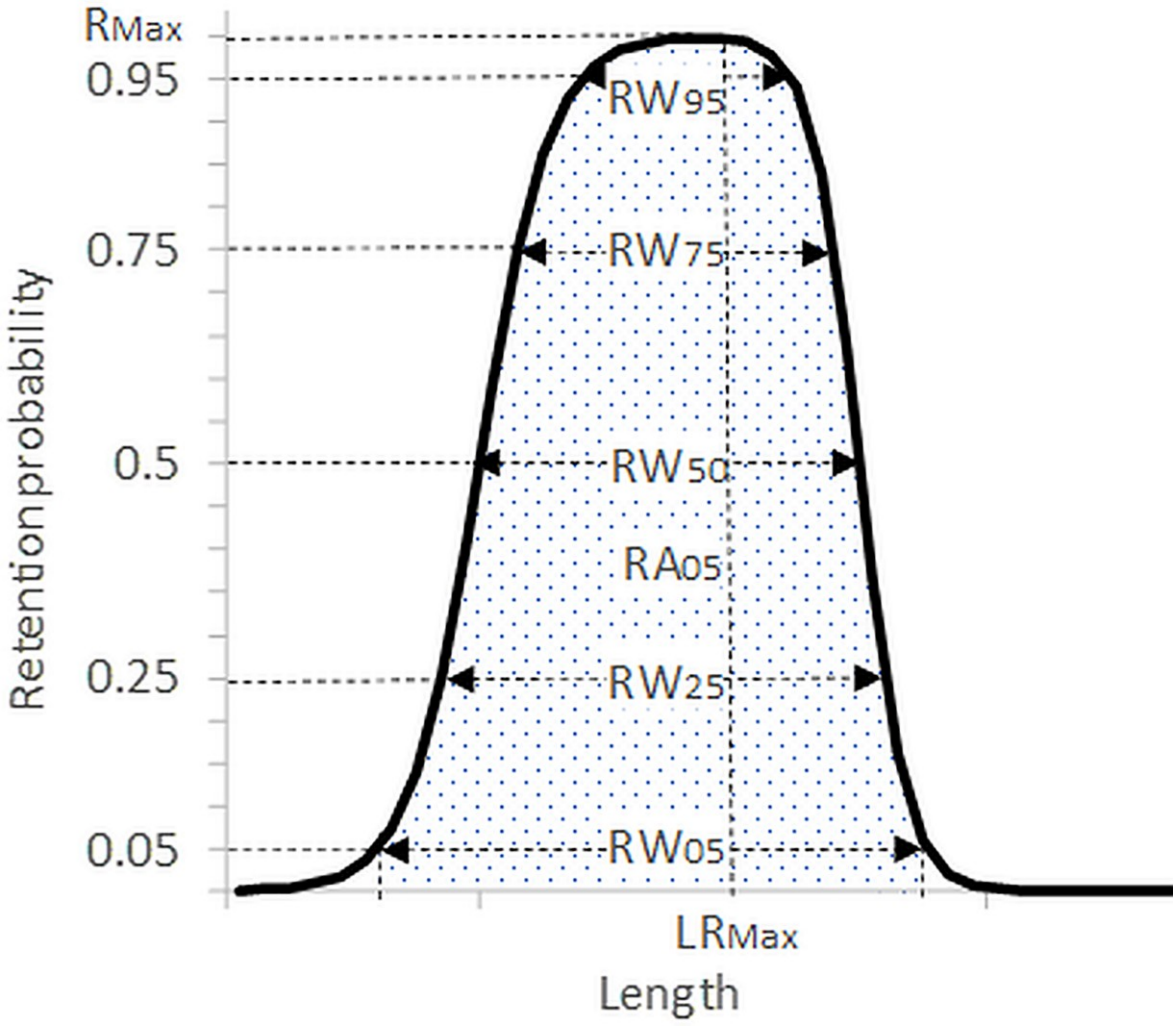
Bell-shaped retention curve with indicators.

Because the bell-shaped retention risk (probability) for the bycatch species as given by *r*_*combined*_(*l*,***υ***_*grid*_,***υ***_*codend*_) is an issue of great importance in fisheries management, indicators related to the bell-shaped retention curve were examined. The indicators *RW*_*05*_, *RW*_*25*_, *RW*_*50*_, *RW*_*75*_, *RW*_*95*_, *R*_*max*_, *LR*_*max*,_ and *RA*_*05*_ are all related to the bell-shaped retention curve (Fig 3) and were calculated using a numerical technique implemented in the software tool SELNET [29].

*RW*_*05*_, *RW*_*25*_, *RW*_*50*_, *RW*_*75*_, and *RW*_*95*_ quantify the length span (in mm for the shrimp and in cm for the rest of the species) with at least 5, 25, 50, 75, and 95% probability of retention, respectively. *R*_*max*_ is the maximum retention probability on the bell-shaped curve, and *LR*_*max*_ is the corresponding shrimp, redfish, or American plaice length (in mm for the shrimp and in cm for the rest of the species). *RA*_*05*_ quantifies the area of the retention bell-shaped curve at which the retention probability is ≥5%. For each of the indicators, 95% confidence limits were estimated using the double bootstrap method described above.

### Inference of difference in codend size selection and combined retention among designs

To infer the effect of changing from one codend to another (e.g. from codend *A* to codend *B*) on codend size selection *r*_*codend*_(*l*,***υ***_*codend*_) and on the combined selection curve *r*_*combined*_(*l*,***υ***_*grid*_,***υ***_*codend*_), the length-dependent change Δ*r*(*l*) (delta in retention rate at length) in the values was estimated by:

$$\Delta r(l)=r_{B}(l)-r_{A}(l)$$ (5)

where *r*_*A*_(*l*) represents the value for *r*_*codend*_(*l*,***υ***_*codend*_) or *r*_*combined*_(*l*,***υ***_*grid*_,***υ***_*codend*_) for the design with codend *A* and *r*_*B*_(*l*) represents the same for the design with codend *B*. Efron 95% percentile [27] confidence limits for Δ*r*(*l*) were obtained based on the two bootstrap populations created (1000 bootstrap repetitions in each) for *r*_*A*_(*l*) and *r*_*B*_(*l*). As they are obtained independently, a new bootstrap population of results was created for Δ*r*(*l*) by:

$${\Delta r(l)}_{i}={r_{B}(l)}_{i}-{r_{A}(l)}_{i}\;i\in [1\ldots 1000]$$ (6)

where *i* denotes the bootstrap repetition index. As the bootstrap resampling was random and independent for the two groups of results, it is valid to generate the bootstrap population of results for the difference based on (6) using the two independently generated bootstrap files [32]. Based on the bootstrap population, Efron 95% percentile confidence limits were obtained for Δ*r*(*l*) as described above.

## Results

### Data collection

The study included a total of 32 hauls: eight carried out with the diamond mesh codend, eight with the diamond mesh codend with square mesh panels, eight with the square mesh sorting cone, and eight control hauls. During the cruise we length-measured a total of 8,418 shrimp, 14,943 American plaice, and 9,418 redfish (Table 1).

**Table 1:** Overview of the hauls collected for the present study. For each haul, towing time (min), depth (m), the position at trawl start (Latitude and Longitude), and the number of length measured shrimp, American plaice and redfish are provided. The numbers in brackets are the subsampling factors provided as percentage for the shrimp catches. GC = grid cover control, GT = grid cover test, CC = control codend, CT = test codend (Fig 2).

Haul No	Codend	Tow time (min)	Depth (m)	LAT	LONG	*Shrimp*		*American Plaice*		*Redfish*	
						GC or GT (% measured)	CC or CT (% measured)	GC or GT	CC or CT	GC or GT	CC or CT
H202	Square	61	267	7605.6N	03523.1E	119 (17.89)	163 (2.23)	268	76	90	16
H204	Control	60	268	7604.9N	03526.9E	123 (72.31)	160 (1.63)	208	177	56	36
H205	Control	61	257	7605.4N	03517.8E	120 (58.14)	153 (1.95)	238	182	143	37
H206	Square	59	277	7606.6N	03533.9E	21 (100.00)	117 (9.16)	39	26	79	8
H207	Square	60	265	7606.0N	03518.2E	185 (42.80)	132 (4.64)	107	75	157	14
H208	Control	60	278	7605.3N	03511.1E	163 (7.47)	173 (1.16)	438	187	404	169
H210	Control	60	271	7605.9N	03533.8E	108 (9.60)	171 (1.20)	265	156	184	86
H211	Square	60	257	7604.5N	03516.4E	133 (31.25)	132 (6.26)	77	79	54	9
H212	Square	61	267	7604.2N	03507.6E	111 (100.00)	157 (2.70)	150	134	136	26
H213	Control	63	266	7605.9N	03521.9E	144 (40.54)	160 (1.91)	321	121	108	20
H214	Control	61	271	7606.5N	03531.9E	169 (100.00)	175 (2.02)	206	150	68	34
H215	Square	62	267	7606.1N	03520.8E	122 (38.76)	139 (1.89)	288	143	107	30
H216	Square	60	285	7607.4N	03533.0E	99 (17.01)	133 (1.95)	215	142	154	30
H217	Control	60	271	7606.6N	03521.9E	208 (22.74)	169 (1.02)	391	287	187	94
H218	Control	63	272	7606.5N	03531.9E	189 (21.12)	190 (0.73)	327	301	164	120
H220	Square	63	276	7606.4N	03532.4E	120 (40.35)	162 (1.04)	252	201	183	56
H221	Diamond	60	268	7606.1N	03522.3E	150 (63.13)	150 (1.34)	391	283	211	42
H225	Diamond	62	265	7605.4N	03523.3E	123 (31.72)	146 (0.94)	444	347	392	65
H226	Diamond	64	268	7605.8N	03525.1E	98 (66.77)	134 (1.05)	482	402	494	108
H229	Diamond	62	265	7605.7N	03522.1E	7 (100.00)	121 (2.10)	283	309	211	47
H230	Diamond	63	274	7605.9N	03523.4E	21 (100.00)	141 (1.76)	239	212	354	91
H233	Diamond	60	256	7604.7N	03516.8E	50 (100.00)	161 (2.67)	256	202	98	33
H234	Diamond	63	252	7604.0N	03512.9E	75 (80.61)	146 (1.08)	230	320	135	82
H238	Diamond	66	269	7606.1N	03517.2E	140 (8.18)	167 (1.78)	298	120	142	24
H244	Sort. Co	60	276	7606.9N	03534.1E	124 (4.98)	162 (1.46)	375	127	572	91
H246	Sort. Co	62	262	7604.7N	03533.9E	140 (10.76)	177 (1.34)	376	197	424	56
H249	Sort. Co	60	261	7605.8N	03521.3E	123 (32.26)	145 (1.42)	441	260	373	47
H250	Sort. Co	30	263	7604.9N	03531.5E	40 (100.00)	148 (7.64)	33	83	124	11
H254	Sort. Co	60	268	7604.6N	03536.4E	154 (32.59)	147 (1.82)	133	109	305	34
H255	Sort. Co	60	266	7605.6N	03523.8E	128 (21.60)	133 (1.24)	428	227	296	35
H258	Sort. Co	62	269	7604.1N	03537.5E	0 (0.00)	156 (1.47)	314	243	704	64
H259	Sort. Co	60	264	7605.0N	03528.4E	36 (100.00)	155 (2.04)	271	281	612	82

The size selectivity of shrimp, American plaice, and redfish for the diamond mesh codend included in this investigation was studied thoroughly by Larsen et al. [4]. Therefore, a detailed examination of the results for this codend was not repeated in this study. However, because the results of the diamond mesh codend are used in the comparisons with the two other codends tested in this study and the indicators for the bell-shaped retention probability were not provided in Larsen et al. [4], herein we provide a new results table for the diamond mesh codend (Table 2).

**Table 2:** Selectivity results [4] and bell-shaped retention probability results obtained for the grid and diamond mesh codend tested during the trials. Confidence intervals for the values are given in brackets. Results for the three species included in the study are given in the table. Shrimp measured in mm, fish in cm.

	DIAMOND MESH CODEND		
	SHRIMP	REDFISH	AMERICAN PLAICE
*Cgrid* (%)	100.00 (98.99–100.00)	90.47 (76.34–99.12)	100 (97.76–100.00)
*L50grid* (mm/cm)	49.17 (38.51–66.81)	13.61 (12.99–14.25)	19.40 (18.36–20.24)
*SRgrid* (mm/cm)	16.52 (8.37–27.70)	3.46 (2.98–4.02)	7.47 (6.45–8.61)
*L50codend* (mm/cm)	17.72 (16.17–22.65)	9.78 (8.95–10.40)	6.84 (5.54–7.81)
*SRcodend* (mm/cm)	3.63 (1.76–8.89)	1.74 (1.32–2.60)	1.66 (0.10–2.52)
*SP*	0.51 (0.42–0.71)	0.63 (0.51–0.73)	0.55 (0.49–0.61)
*Rmax* (%)	95.02 (84.74–98.51)	63.89 (52.69–73.95)	92.76 (88.45–100.00)
*LRmax* (mm/cm)	25.51 (21.49–34.80)	11.51 (11.04–12.06)	10.23 (6.49–11.67)
*RW05* (mm/cm)	37.16 (34.76–40.72)	10.70 (9.73–11.89)	24.96 (23.02–26.39)
*RW25* (mm/cm)	34.08 (25.05–35.71)	6.07 (5.40–7.13)	17.12 (16.01–18.40)
*RW50* (mm/cm)	31.40 (17.71–33.56)	3.02 (1.35–4.12)	12.53 (11.32–13.92)
*RW75* (mm/cm)	21.20 (10.21–31.13)	0.00 (0.00–0.00)	7.82 (6.30–9.64)
*RW95* (mm/cm)	0.00 (0.00–14.83)	0.00 (0.00–0.00)	0.00 (0.00–2.68)
*RA05* (mm/cm)	1.86 (1.96–29.36)	3.51 (2.95–4.25)	12.44 (11.34–13.77)

### Selectivity of the diamond mesh codend with square mesh panels

**Fig 4:**
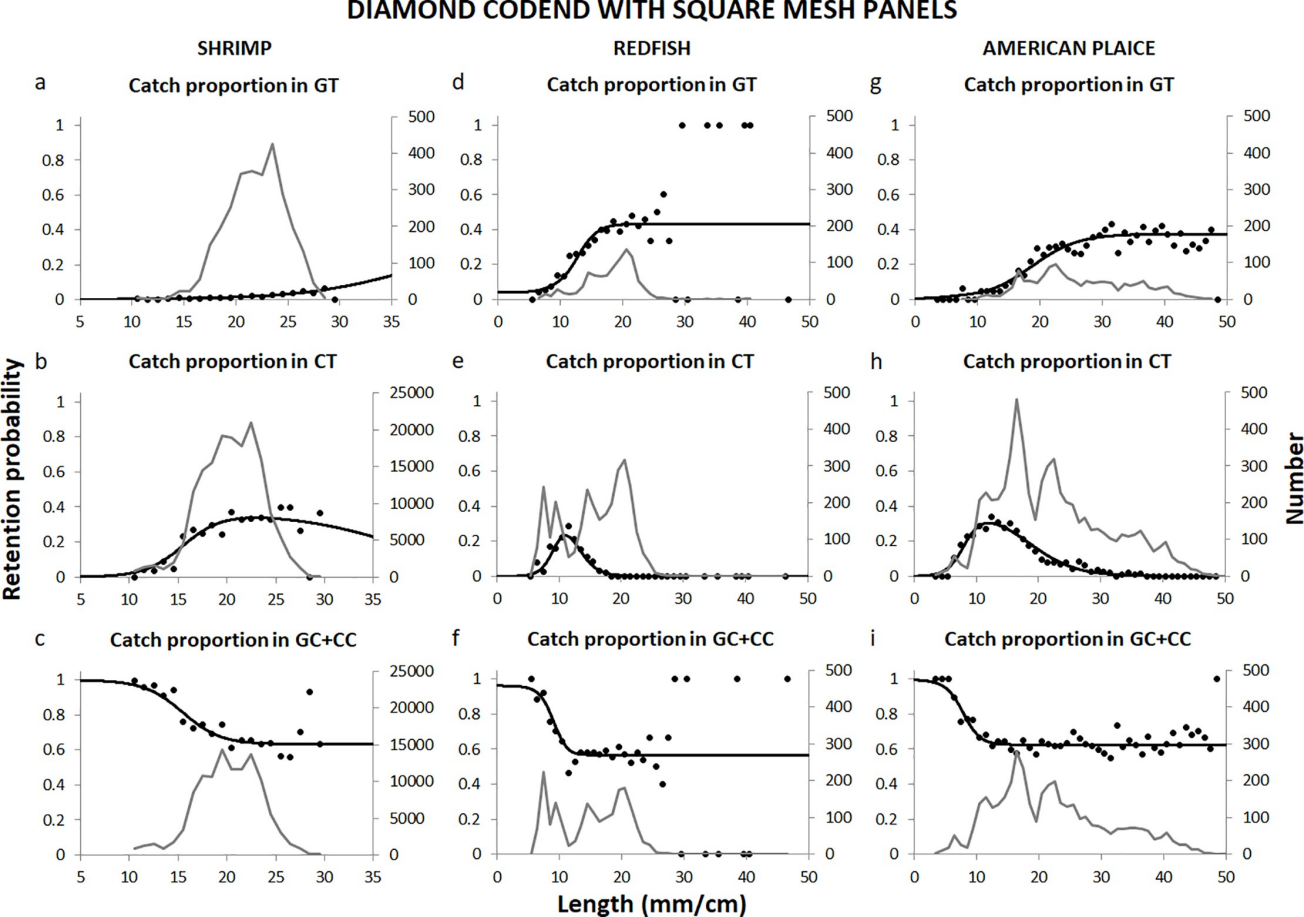
Catch proportion observed with the grid and diamond mesh codend with square meshes system. The test grid cover (GT), test codend (CT) and control grid cover + control codend (GC + CC), the model fit and size distribution (grey line) for the shrimp (a-c), redfish (d-f) and American plaice (g-i). Note that the catch distribution presented for the shrimp is based on raised numbers according to the subsampling factors (Table 1).

The size-selectivity results obtained with the diamond mesh codend with square mesh panels added showed that the model used fitted the experimental data well for all three species (Fig 4). For the shrimp, the p-value was low, but this was most likely a consequence of over-dispersion in the experimental catch portioning data that resulted from working with pooled and subsampled data with low sampling rates (Table 1). The p-values estimated for redfish and American plaice (0.4338 and 0.1583, respectively) and the deviance vs. DOF confirm the good fit observed in Fig 4. The contact probability with the grid was high and estimated to be above 94% for all three species (Table 3). The indicators show that even with the square meshes in the codend, the retention of redfish was still ~50% for redfish of around 11 cm and ~79% for American plaice around 12 cm. Furthermore, the differences in the estimated *R*_*max*_ obtained with this codend versus the diamond mesh codend were not significant for either species. For the shrimp, the maximum retention obtained with the grid and the diamond mesh codend with square meshes was ~91% for shrimp of around 24 mm in carapace length.

**Table 3:** Selectivity results obtained for grid and the diamond mesh codend with square meshes system tested during the trials. Confidence intervals for the values are given in brackets. Results for the three species included in the study are given in the table. Shrimp measured in mm, fish in cm.

	DIAMOND CODEND WITH SQUARE MESH PANELS		
	SHRIMP	REDFISH	AMERICAN PLAICE
*Cgrid* (%)	100.00 (97.70–100.00)	94.86 (70.55–100.00)	100.00 (98.28–100.00)
*L50grid* (mm/cm)	37.73 (32.76–51.55)	12.55 (11.48–14.53)	18.60 (17.40–19.94)
*SRgrid* (mm/cm)	11.91 (6.50–20.49)	3.58 (2.14–4.22)	8.17 (6.69–10.03)
*L50codend* (mm/cm)	16.41 (12.61–48.57)	9.99 (8.62–11.80)	8.65 (6.49–10.89)
*SRcodend* (mm/cm)	4.28 (0.10–18.53)	2.40 (1.47–9.83)	2.95 (0.10–6.75)
*SP*	0.37 (0.26–0.95)	0.43 (0.34–0.55)	0.37 (0.28–0.45)
*Rmax* (%)	90.85 (20.84–100.00)	49.87 (35.08–62.40)	79.36 (59.93–100.00)
*LRmax* (mm/cm)	23.61 (13.50–42.32)	11.39 (10.46–12.41)	12.44 (7.49–14.71)
*RW05* (mm/cm)	39.37 (20.89–43.43)	10.51 (9.02–15.62)	24.92 (21.97–27.82)
*RW25* (mm/cm)	29.42 (0.00–37.01)	5.18 (4.01–7.23)	15.47 (13.93–17.14)
*RW50* (mm/cm)	21.24 (0.00–34.52)	0.00 (0.00–2.85)	9.75 (6.51–12.25)
*RW75* (mm/cm)	12.94 (0.00–25.10)	0.00 (0.00–0.00)	3.31 (0.00–8.16)
*RW95* (mm/cm)	0.00 (0.00–8.80)	0.00 (0.00–0.00)	0.00 (0.00–1.27)
*RA05* (mm/cm)	1.97 (2.86–28.93)	2.74 (2.14–3.76)	9.99 (8.09–12.13)
p-value	<0.0001	0.4338	0.1583
Deviance	114.84	59.14	101.24
DOF	34	58	88

### Selectivity of the sorting cone

**Fig 5:**
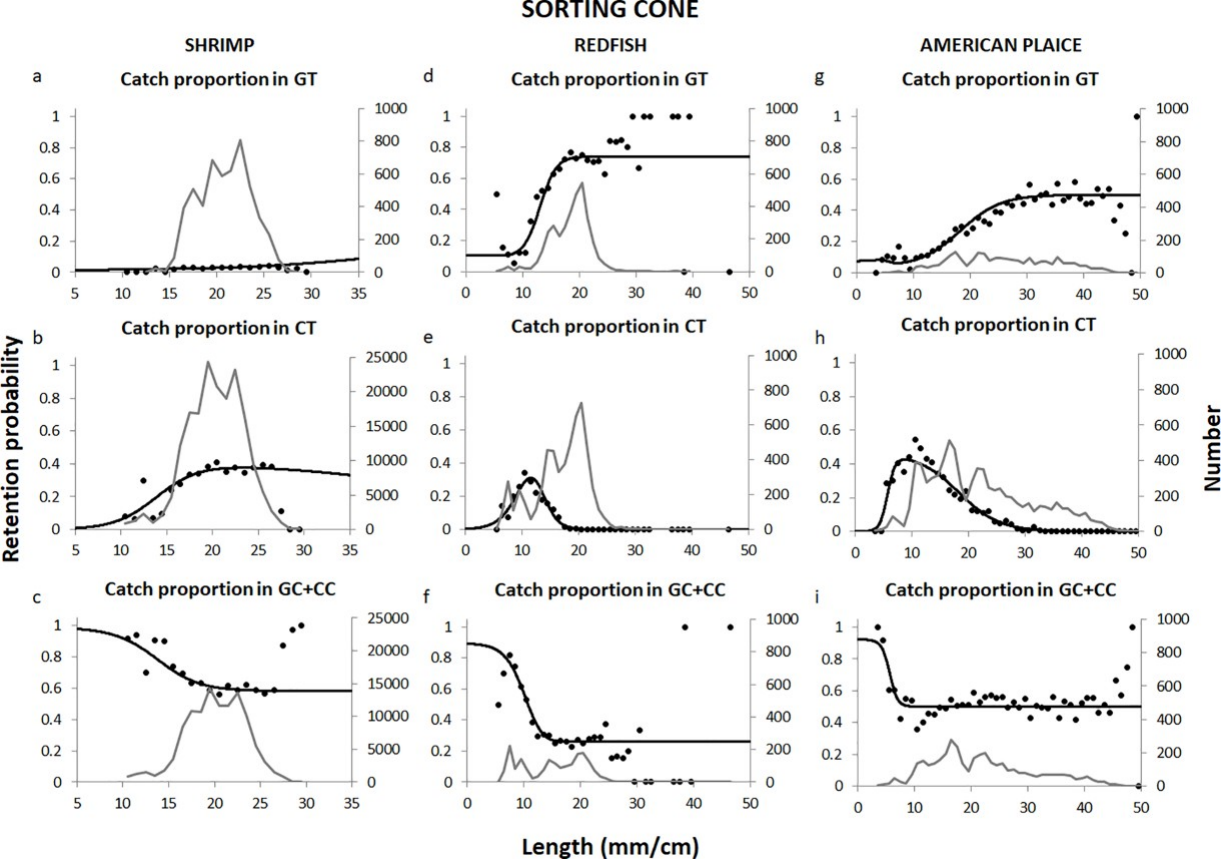
Catch proportion observed with the grid and sorting cone system. Test grid cover (GT), test codend (CT) and control grid cover + control codend (GC + CC), the model fit and size distribution (grey line) for the shrimp (a-c), redfish (d-f) and American plaice (g-i). Note that the catch distribution presented for the shrimp is based on raised numbers according to the subsampling factors (Table 1).

The plots in Fig 5 show that the model applied to the experimental data represented the data well for all three species. Therefore, even though the p-value was low for all three species (especially for shrimp and American plaice), we are confident that the model chosen was adequate. For the shrimp, the low p-value occurred for the same reason explained above for the diamond mesh codend with square mesh panels. For American plaice, the low p-value was the result of unequal entry of bigger American plaice into the test and control gears. However, this discrepancy was only observed for fish above 40 cm, which is well above the selective range of the gear used (Fig 5G–5I). A similar phenomenon was observed for redfish above 25 cm, which again is outside the selective range of the gear for this species (Fig 5D–5F).

Contact with the grid was high and estimated to be above 90% in all cases (Table 4). The indicators show that the maximum retention for redfish with this system was ~28% for fish of 13 cm, which is significantly lower than the *R*_*max*_ obtained with the diamond mesh codend. For American plaice, on the other hand, the maximum retention was observed for smaller fish (9 cm) but was much higher than for redfish (~86%). For shrimp, the maximum retention and size obtained with this system were very similar to those obtained with the grid and diamond mesh codend with square mesh panel system.

**Table 4:** Selectivity results obtained for grid and sorting cone system tested during the trials. Confidence intervals for the values are given in brackets. Results for the three species included in the study are given in the table. Shrimp measured in mm, fish in cm. Note that *L50*_*codend*_ and *SR*_*codend*_ in this case refer to the sorting cone even though the shrimp and bycatch fish were retained in the blinded codend installed subsequent to the cone.

	SORTING CONE		
	SHRIMP	REDFISH	AMERICAN PLAICE
*Cgrid* (%)	100.00 (92.22–100.00)	95.91 (86.73–100.00)	92.35 (82.34–100.00)
*L50grid* (mm/cm)	50.16 (29.02–185.77)	13.45 (12.72–14.28)	18.26 (16.99–19.52)
*SRgrid* (mm/cm)	24.65 (0.10–100.00)	2.73 (2.45–2.99)	7.89 (6.81–9.06)
*L50codend* (mm/cm)		15.12 (10.57–30.22)	13.03 (11.20–16.13)	6.10 (5.03–7.34)
*SRcodend* (mm/cm)		5.01 (0.10–20.98)	4.37 (2.55–10.56)	1.48 (0.10–2.10)
*SP*	0.42 (0.33–0.67)	0.74 (0.64–0.82)	0.50 (0.40–0.57)
*Rmax* (%)	89.34 (68.15–100.00)	28.44 (20.42–41.23)	85.75 (77.55–100.00)
*LRmax* (mm/cm)	24.25 (12.50–40.00)	12.64 (11.62–13.37)	9.19 (6.48–10.47)
*RW05* (mm/cm)	31.58 (18.42–37.00)	9.66 (8.24–14.79)	24.45 (21.78–25.91)
*RW25* (mm/cm)	27.30 (13.67–32.45)	2.10 (0.00–3.99)	16.44 (14.89–17.66)
*RW50* (mm/cm)	24.67 (8.64–28.62)	0.00 (0.00–0.00)	11.36 (9.79–13.11)
*RW75* (mm/cm)	19.61 (0.00–28.18)	0.00 (0.00–0.00)	5.60 (2.46–8.55)
*RW95* (mm/cm)	0.00 (0.00–3.35)	0.00 (0.00–0.00)	0.00 (0.00–0.56)
*RA05* (mm/cm)	1.58 (1.66–24.91)	1.62 (1.24–2.36)	11.12 (9.69–12.84)
p-value	<0.0001	0.0068	<0.0001
Deviance	184.89	90.43	165.64
DOF	34	60	90

### Comparisons with the diamond mesh codend

We compared the selectivity of the two new systems tested in the present study with the compulsory system consisting of the Nordmøre grid and a 35-mm diamond mesh codend to investigate whether adding square mesh panels in the codend or substituting the diamond mesh codend with a sorting cone could improve the selectivity of the gear for shrimp, redfish, or American plaice.

**Fig 6:**
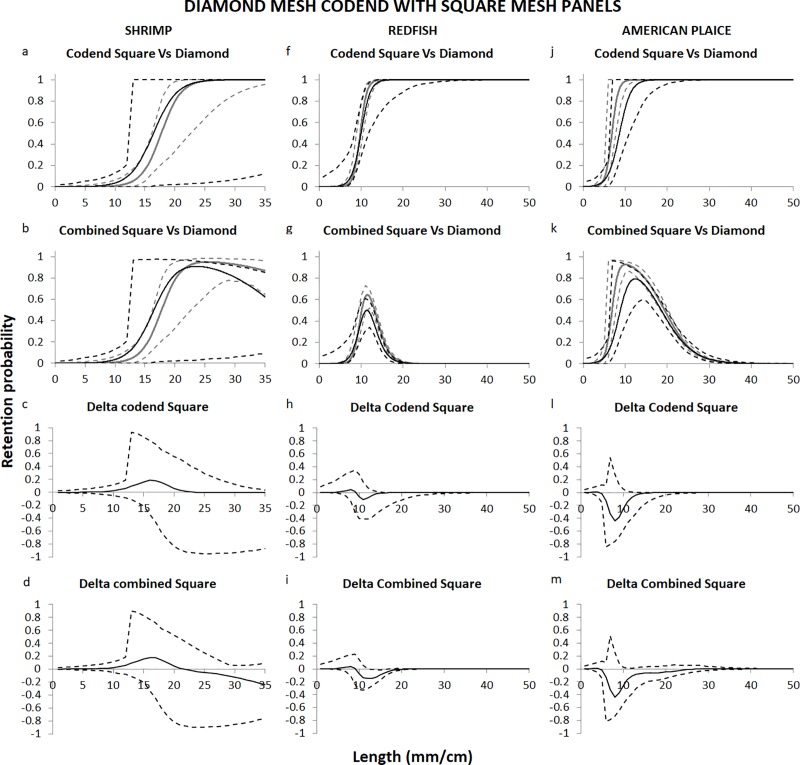
**Codend and combined (grid and codend) selectivity curves and delta plots according to Eq (5) obtained with the diamond mesh codend (grey) and the diamond mesh codend with square meshes (black).** Shrimp (a-b, c-d), redfish (f-g, h-i) and American plaice (j-k, l-m). Dashed lines represent the 95% confidence intervals for the curves.

Changing the diamond mesh codend to a diamond mesh codend with square mesh panels did not result in any major differences in the selectivity properties of the gear (see delta plots in Fig 6). The only marked improvement was for redfish, and when the selectivity of the grid and the codend combined were considered, the differences between the two systems become significant for sizes between ca. 13 and 19 cm (Fig 6I). For American plaice, the square mesh panels did not contribute to additional release of fish (Fig 6L and 6M). According to these results, the square mesh panels in the codend did not contribute to the release of any additional undersized shrimp (< 15 mm carapace length) (Fig 6C and 6D).

**Fig 7:**
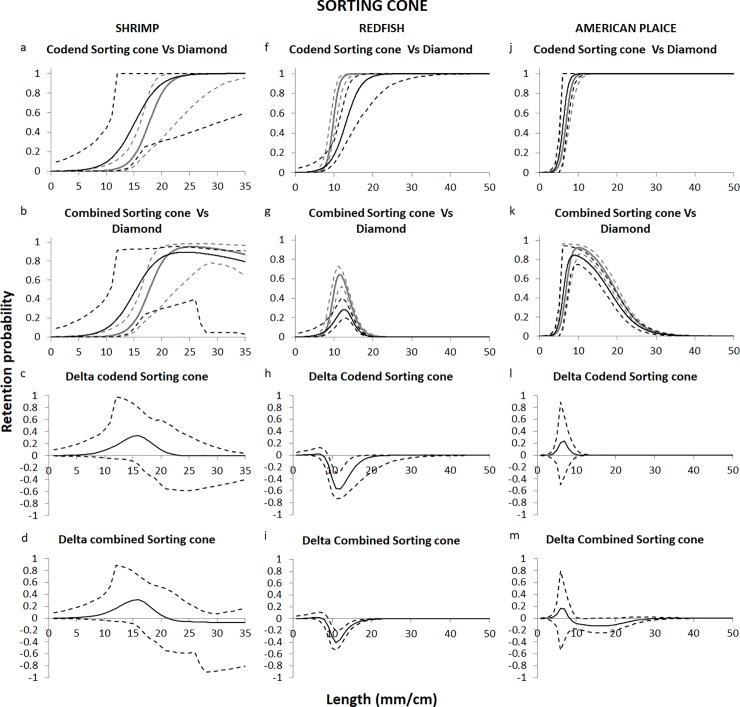
**Codend and combined (grid and codend) selectivity curves and delta plots according to Eq (5) obtained with the diamond mesh codend (grey) and the sorting cone (black).** Shrimp (a-b, c-d), redfish (f-g, h-i) and American plaice (j-k, l-m). Dashed lines represent the 95% confidence intervals for the curves.

For shrimp, substituting the diamond mesh codend with a sorting cone did not result in any significant change in the retention probability of the different length classes captured (Fig 7A–7D). For American plaice, differences between the two systems were only noticeable when the combined grid and codend selectivity curves were compared. In this case, the differences between the systems were not very pronounced (Fig 7M). For redfish, the differences between the diamond mesh codend and the sorting cone were much clearer, either when combined or not combined with the grid. In both cases, the system with the sorting cone retained significantly fewer redfish between approximately 9 and 18 cm (Fig 7H and 7I).

## Discussion

In the present study, we investigated whether the selectivity of the size selection gear used by the shrimp trawlers fishing in the Barents Sea, a 19-mm bar-spacing Nordmøre grid combined with a 35-mm diamond mesh codend, could be improved by two alternative codends: a diamond mesh codend with square mesh panels or a codend with a square mesh sorting cone. The results of our experiments show that using square mesh panels at the bottom and sides of a diamond mesh codend did not provide any additional release of undersized shrimp or American plaice. For American plaice this is in line with the expectation as the morphology of a flatfish is not better suited for escape through a square mesh [22, 23, 24]. Contrary, for redfish, we would have expected an increase in release rate, but the results do not show any clear improvement in release. Studies of the performance of square mesh panels or codends are very scarce in the Barents Sea shrimp fishery or comparable fisheries that involve the same species. Thorsteinsson [19] carried out two cruises in the Icelandic coastal shrimp fishery to compare the performance of two square mesh codends with mesh sizes of 36 and 40 mm with that of diamond mesh codends of 36- and 37-mm mesh sizes. He found that there was a loss of shrimp of marketable sizes but that the dramatic reduction in undersized shrimp and juvenile fish bycatch compensated for the loss. In contrast, Lehmann et al. [33] did not detect significant differences in shrimp catch distribution between a 45-mm diamond mesh codend and a 45-mm square mesh codend. In studies conducted in other fisheries around the world, square mesh panels/codends have proved to be very efficient at reducing bycatch of fish juveniles without any substantial loss of shrimps [20, 21, 34]. In the present study, we did not obtain similar results for the Barents Sea shrimp trawl fishery. However, the codend tested in this study had square meshes only in a limited part of the codend and in a position different from that used by Broadhurst et al. [20], for example, who reported reductions of 35–40% of small fish in prawn trawl bycatch with no reduction of target catches in an Australian ocean prawn fishery.

There were no published reports about the performance of a codend with a square mesh sorting cone prior to our study. The idea (originally presented by Valdermarsen [35]) behind this device is that it would allow fish to escape though the square meshes in the narrowing cone while shrimp would just flow towards the codend due to their limited swimming ability. We found that the sorting cone was significantly more efficient than the ordinary diamond mesh codend at releasing redfish between 9 and 18 cm in length without any significant loss of shrimp. Contrary to for the codend with the square mesh panels, this result was in line with the expectation of redfish being more suited to escape through square meshes than diamond meshes. The difference in the performance between the codend with the square mesh panels and the sorting cone for redfish, can probably be related to that the latter is a tapered section, which better facilitates contact with the meshes. For American plaice the results obtained with the sorting cone were similar to those obtained for the codend with the square mesh panels, which was in line with the expectation that square meshes would not increase the release rate of American plaice [22, 23, 24]. From our results, it is clear that square meshes do not represent an equally good alternative for American plaice as for redfish. This result is likely related to the morphological characteristics of flatfish, whose shape is better fitted to diamond mesh with a low opening angle than a square mesh of the same size.

The towing time and trawls used in this experiment were smaller than those used by the commercial vessels, meaning that the catches obtained were lower than those typically obtained by the fleet. Further, even though there was no indication that the covers affected the performance of the selective devices tested, commercial vessels do not use small-meshed covers to capture escapees from the grid section. Therefore, some precaution needs to be taken regarding extrapolation of the results presented here to commercial fishing conditions. However, except for the points mentioned, we carried out the experiments following commercial practice and we assume that the selective devices tested would perform similar under commercial fishing.

The impact of fishing gear selectivity relies on the assumption that most animals escaping the fishing gear survive. This assumption is not necessarily always fulfilled, and a certain percentage of the escapees may perish due to the damages perceived when passing through the gear. Unaccounted fishing mortality can lead to underestimation of the positive impact of fishing gear selectivity on the fish stocks. Therefore, escapee survival studies are of great value. To our knowledge, no study has investigated the survival of redfish, American plaice or shrimp juveniles escaping from a shrimp trawl in the Barents Sea. However, earlier survival studies carried out in adjacent areas have shown high survival rates for redfish [36], American plaice [37] and shrimp [38] escaping through codend meshes, which should encourage further selectivity work in the Barents despite the need for further survival studies in the area.

In general, the results obtained in this study show that the model applied by Larsen et al. [4] is indeed suitable for selectivity data that include two selection processes and produce retention data that follow a bell-shaped pattern. This is clearly demonstrated by the model fit to the data shown in Figs 4 and 5. In the future, experiments using the sorting cone should be conducted and applied to other bycatch species, considering the promising results obtained for redfish in the present study. Taking into account that the sorting cone could also be inserted in the gear as an additional device in front of the codend, it would be interesting to see if it could contribute to the selectivity of specific codends. The results for the square mesh panels combined with a diamond mesh codend were not as encouraging as expected. Nevertheless, considering the results obtained in other shrimp/prawn fisheries [20, 21, 34] and by previous trials in the Northeast Atlantic [19], square mesh panels placed in other positions in the codend or codends constructed entirely of square meshes would be worth testing.

## Supporting information

S1 Catch Data for individual hauls. The catch data consist of count data for number shrimp, redfish and American plaice caught with respectively the control codend, diamond mesh codend, diamond mesh codend with square mesh panels and sorting cone codend for each size class (Length). For redfish and American plaice “Length” corresponds to the total length of the fish whereas for shrimp it corresponds to the carapace length.
(ZIP)
